# Validation of the Korean version of the Self-Dehumanization Scale

**DOI:** 10.3389/fpsyt.2026.1754166

**Published:** 2026-03-05

**Authors:** Khongorzul Khosbayar, Jang-won Seo

**Affiliations:** Department of Psychology, Jeonbuk National University, Jeonju, Republic of Korea

**Keywords:** reliability, SDS, self-dehumanization, Self-Dehumanization Scale, validity

## Abstract

**Introduction:**

Self-dehumanization refers to the perception of oneself as less human, and it has been closely associated with psychological difficulties such as depression, suicidal ideation, and self-hatred. The Self-Dehumanization Scale (SDS) was developed to assess animalistic and mechanistic forms of self-dehumanization.

**Method:**

This study aimed to translate and validate a Korean version of the SDS (K-SDS) in an adult sample. An online survey was conducted with Korean adults aged 19 years and older (*N* = 844). We assessed internal consistency of the K-SDS and performed confirmatory factor analysis to determine the adequacy of the original two-factor structure.

**Results:**

Convergent and discriminant validity of the scale were assessed through correlations with measures of depression, suicidal ideation, self-hatred, and perceived burdensomeness and thwarted belongingness. Measurement invariance was tested across gender, age, and education groups, and latent mean analyses were performed for gender and education levels. Results indicated that the K-SDS demonstrated good internal consistency and that the two-factor structure provided the best model fit. Convergent and discriminant validity were generally supported, with animalistic self-dehumanization showing stronger associations with depression and suicidal ideation. Measurement invariance was established across groups, and latent mean analyses revealed that men reported higher levels of self-dehumanization than women, while individuals with higher education reported significantly lower levels.

**Discussion:**

The Korean version of the Self-Dehumanization Scale (K-SDS) demonstrates reliable and valid psychometric properties and may serve as a promising instrument for assessing self-dehumanization in Korean adults.

## 1 Introduction

Humans exhibit advanced cognitive abilities such as intelligence, moral reasoning, and higher-order functions. These attributes underlie social bonding and facilitate adaptation to diverse environmental conditions. However, these qualities are disregarded or denied in certain situations, which leads individuals to be perceived as animalistic or mechanistic, a process known as dehumanization (1). When internalized, such perceptions can have significant psychological consequences (2, 3).

Dehumanization is a social and historical process wherein specific individuals or groups are perceived as lacking essential human attributes, such as intelligence, morality, and maturity, and are consequently treated as less than humans. These perceptions are often used to rationalize discrimination, abuse, and mass violence (4). Beyond perceptual distortion, dehumanization functions as a psychological mechanism that allows perpetrators to justify their violent and oppressive behaviors without guilt (5). Although initial research predominantly investigated extreme scenarios such as genocide and sustained intergroup conflict (6, 7), subsequent studies emphasized the identification of dehumanization occurring in more subtle ways in everyday situations, such as experiences of neglect and abuse (8, 9). Despite the variability in presentation depending on the severity of the context, dehumanization consistently involves the denial of the human essence.

More importantly, dehumanization not only occurs at the level where others are perceived as less human but it can also occur at a level where it is internalized by individuals, leading them to perceive themselves as less human—a phenomenon termed *self-dehumanization* (10). Individuals may internalize these perceptions when they are treated without humanity, which may exacerbate their psychological distress (2). Unlike general negative self-perceptions, such as self-hatred or low self-esteem, self-dehumanization explicitly involves denying fundamental human qualities, including agency, emotional warmth, civility, and morality (11, 12).

Self-dehumanization can be understood through Haslam’s (1) two-dimensional model of humanness, which separates human nature (HN; attributes such as emotionality, agency, and cognitive flexibility that distinguish humans from objects) and human uniqueness (HU; attributes such as civility, refinement, and morality that distinguish humans from animals). Individuals who deny HN may perceive themselves as cold, rigid, or mechanical. In contrast, individuals who deny HU may view themselves as immature, coarse, or animal-like (9). Self-dehumanization can manifest in two forms: mechanistic self-dehumanization (denial of human nature, HN) and animalistic self-dehumanization (denial of human uniqueness, HU).

Recent research has emphasized that self-dehumanization is a key risk factor for psychological difficulties, including depressive symptoms and suicidal ideation (13–16). Additionally, it functions distinctly from related concepts such as dissociation (17) and offers a more precise understanding of existing suicide risk factors such as perceived burdensomeness, i.e., the belief that one’s existence imposes a burden on others, and thwarted belongingness, i.e., a sense of social disconnection or exclusion (18, 19). However, despite its clinical significance, a standardized measure to assess self-dehumanization has been lacking until recently.

Therefore, Robison et al. (20) developed the Self-Dehumanization Scale (SDS) by integrating Haslam’s (1) theoretical model of humanness with empirical research on dehumanization to address the literature gap. The SDS operationalizes self-dehumanization in terms of animalistic and mechanistic dimensions, and has been validated in three studies. The final version comprises eight items loaded on two stable factors, demonstrating good internal consistency and construct validity. Notably, self-dehumanization is associated with depressive symptoms, suicidal ideation, perceived burdensomeness, thwarted belongingness, self-hatred, and experiences of social discrimination, highlighting its distinct clinical significance.

Thus, the present study adapted and validated the SDS for use in Korea, where suicide rates are the highest among OECD countries and cultural factors such as self-criticism and social isolation can increase vulnerability to self-dehumanizing thoughts (21). Specifically, we examined the reliability, factor structure, convergent validity, discriminant validity, and criterion validity of the instrument along with measurement invariance across sex and age groups. Establishing validity is essential to ensure the cross-cultural applicability of the SDS and enable an accurate assessment of self-dehumanization in clinical and research contexts in Korea.

## 2 Method

### 2.1 Participants

We recruited 900 Korean adults aged 18–76 years across the country through a professional online survey company. After excluding 56 multivariate outliers based on the Mahalanobis distance and z-scores (*p* <.001), the final sample included 844 participants (418 men, 49.5%; 426 women, 50.5%). The participants’ ages ranged from 18 to 75 years (M = 45.2, SD = 15.4). Participants were categorized into three age groups based on the Erikson’s (22) psychosocial developmental theory: 18–29 years (n = 271), 30–49 years (n = 282), and 50–75 years (n = 291). Educational level was categorized into three groups: lower (n = 268), middle (n = 458), and higher (n = 118). All participants provided written informed consent before participation. The Institutional Review Board of Jeonbuk National University approved the present study (IRB No. 2025-01-010).

### 2.2 Procedure

Participants completed an online survey that included the K-SDS and other self-report instruments measuring depression, suicidal ideation, and self-hatred. The survey took approximately 15 minutes to complete and was administered anonymously. Written informed consent was obtained from all participants.

### 2.3 Measures

#### 2.3.1 Self-Dehumanization Scale

The SDS (20) is an 8-item self-report measure that assesses the extent to which individuals perceive themselves as lacking essential human qualities, reflecting animalistic and mechanistic forms of self-dehumanization. Items are rated on a 7-point Likert scale (1 = not at all true of me to 7 = very true of me), with higher scores indicating higher self-dehumanization. Previous research has shown that self-dehumanization is associated with depressive symptoms, suicidal thoughts, and self-hatred (20). The first author translated the original version of the Self-Dehumanization Scale (SDS) into Korean. Thereafter, the back-translated version was reviewed and revised by a bilingual psychologist. The back-translated version was reviewed for conceptual equivalence, and discrepancies were resolved through discussion among the authors to ensure linguistic and cultural appropriateness. (see Appendix).

#### 2.3.2 Forms of Self-Criticism and Reassurance Scale – Hated Self

The Forms of Self-Criticism and Reassurance Scale – Hated Self (FSCRS-HS (23)) assesses the degree of self-blame and self-hatred. The Korean version was validated by Cho (24). The subscale includes three items rated on a 5-point Likert scale ranging from 0 (not at all true of me) to 4 (very true of me), with higher scores indicating stronger self-critical tendencies. In the current study, the internal consistency was acceptable (Cronbach’s α = .78).

#### 2.3.3 Acquired Capability for Suicide Scale – Fearlessness About Death

The ACSS-FAD (25) measures fearlessness regarding death, which is a central component of acquired capability for suicide. Seo and Kwon (26) developed and validated a Korean version of this scale. The measure comprises seven items rated on a 5-point Likert scale ranging from 0 (not at all like me) to 4 (very much like me), with higher scores indicating a greater fearlessness about death. In the present study, the internal consistency was satisfactory (Cronbach’s α = .84).

#### 2.3.4 Depressive Symptom Index – Suicidality Subscale

The DSI-SS (27) assesses suicidal thoughts and impulses. A Korean version was validated by Suh et al. (28). The scale consists of four items rated on a 4-point Likert scale (0 = not at all to 3 = nearly *every day*), with higher scores reflecting greater suicidality. In the present sample, internal consistency was excellent (Cronbach’s α = .92).

#### 2.3.5 Patient Health Questionnaire-9

The PHQ-9 (29) is a 9-item self-report measure of depressive symptoms based on the DSM diagnostic criteria. Park et al. (30) established a standardized Korean version of this scale. Items are rated on a 4-point Likert scale (0 = not at all to 3 = nearly every day), reflecting the frequency of symptoms over the past two weeks. In the current study, the internal consistency was satisfactory (Cronbach’s α = .87).

#### 2.3.6 Interpersonal Needs Questionnaire-10

The INQ-10 (31) assesses thwarted belongingness and perceived burdensomeness, two key constructs in the interpersonal theory of suicide. Seo (32) validated the Korean version of this scale. The instrument includes ten items rated on a 7-point Likert scale (1 = not at all true of me to 7 = very true of me). In the present study, reliability was strong for both subscales (thwarted belongingness: Cronbach’s α = .84; perceived burdensomeness: Cronbach’s α = .95) and for the total scale (Cronbach’s α = .90).

### 2.4 Statistical analysis

All statistical analyses were conducted using SPSS 27.0 and AMOS 21.0. Additional bifactor confirmatory factor analysis was conducted using the lavaan package in R. The factorial validity of the K-SDS was evaluated through confirmatory factor analysis (CFA) based on the hypothesized two-factor model. Model adequacy was assessed with chi-square (χ²), the root mean square error of approximation (RMSEA), the standardized root mean square residual (SRMR), and the comparative fit index (CFI). According to conventional criteria (33, 34), RMSEA values ≤.05 and SRMR values ≤.05 indicate good fit, values between.05 and.08 indicate acceptable fit, and values up to.10 are considered marginal. For CFI and TLI, values ≥.90 reflect acceptable fit, and values ≥.95 indicate excellent fit (33). All statistical tests were evaluated using a two-tailed significance level of α <.05.

Additionally, we reported the Tucker–Lewis Index (TLI), goodness-of-fit index (GFI), and adjusted goodness-of-fit index (AGFI) to provide additional evidence of model adequacy. Values of GFI ≥.95 and AGFI ≥.90 were indicative of good fit (35).

Measurement invariance was tested across sex, age, and education groups using multi-group CFA. Configural, metric, scalar, and residual invariances were examined sequentially. Invariance was evaluated using chi-square difference tests as well as practical fit indices, with ΔCFI ≤.01 and ΔRMSEA ≤.015 considered evidence of invariance (36, 37). Latent mean differences were then estimated with the reference group mean fixed at zero. Effect sizes for group differences were calculated using Cohen’s *d*.

Finally, the reliability of the K-SDS was assessed with Cronbach’s α, McDonald’s ω, and composite reliability (CR). The relationships between the K-SDS and other measures used in the current study were analyzed through two-tailed Pearson correlations.

## 3 Results

### 3.1 Reliability of the K-SDS

Basic psychometric properties of the K-SDS items are presented in **Table 1**. Skewness ranged from 0.19 to 2.03 and kurtosis ranged from −1.06 to 3.43. Although one item demonstrated elevated kurtosis, overall distributional values were considered acceptable for maximum likelihood estimation given the large sample size. The internal consistency of the total K-SDS scale was good (Cronbach’s *α* = .84; McDonald’s *ω* = .89). The animalistic and mechanistic self-dehumanization subscales also demonstrated acceptable to good internal consistency (Cronbach’s *α* = .75–.89; McDonald’s *ω* = .76–.89).

**Table 1:** Psychometric properties of the K-SDS items (N = 844).

Subscale/item	*M*	*SD*	Skewness	*Kurtosis*
Animalistic self-dehumanization	7.27	4.00		
3- I am socially isolated because I feel less than human	1.98	1.31	1.266	0.622
7- I am disgusted by my lack of humanness	2.04	1.22	1.066	0.296
8- I am a monster	1.50	0.95	2.030	3.433
5- I am less evolved than most other humans	1.75	1.12	1.417	0.910
Mechanistic self-dehumanization	8.22	4.04		
2- I consider myself to be an inanimate object	1.77	1.06	1.367	1.034
1- I view myself as a number	3.09	1.56	0.189	-1.063
6- I sometimes consider myself as an automaton	2.41	1.35	0.665	-0.604
4- I sometimes feel mechanical and cold, like a robot	2.38	1.44	0.797	-0.481
Total scale	16.91	7.57		

M, Mean; SD, Standard Deviation; *α*, Cronbach’s alpha; *ω*, McDonald’s omega.

### 3.2 Factor structure

We performed a confirmatory factor analysis (CFA) to evaluate three competing models of the Korean version of the Self-Dehumanization Scale (K-SDS): a one-factor model, higher-order model, and correlated two-factor model. As shown in **Table 2**, both the one-factor and higher-order models demonstrated an inadequate fit and were not retained.

**Table 2:** Model fit indices for the Korean Self-Dehumanization Scale (K-SDS).

Model	χ²	*df*	TLI	CFI	RMSEA	SRMR
K-SDS/Two-factor	111.17	17	.960	.976	.081	.030
K-SDS/Higher-order	255.21	19	.910	.939	.212	.438
K-SDS/One-factor	258.92	14	.901	.934	.144	.049
K-SDS/Bifactor	64.33	12	.969	.987	.072	.021

*Df*, degrees of freedom; CFI, Comparative Fit Index; TLI, Tucker–Lewis Index; RMSEA, Root Mean Square Error of Approximation; SRMR, Standardized Root Mean Square Residual; All χ² values were significant at *p* <.00.

**Figure 1:**
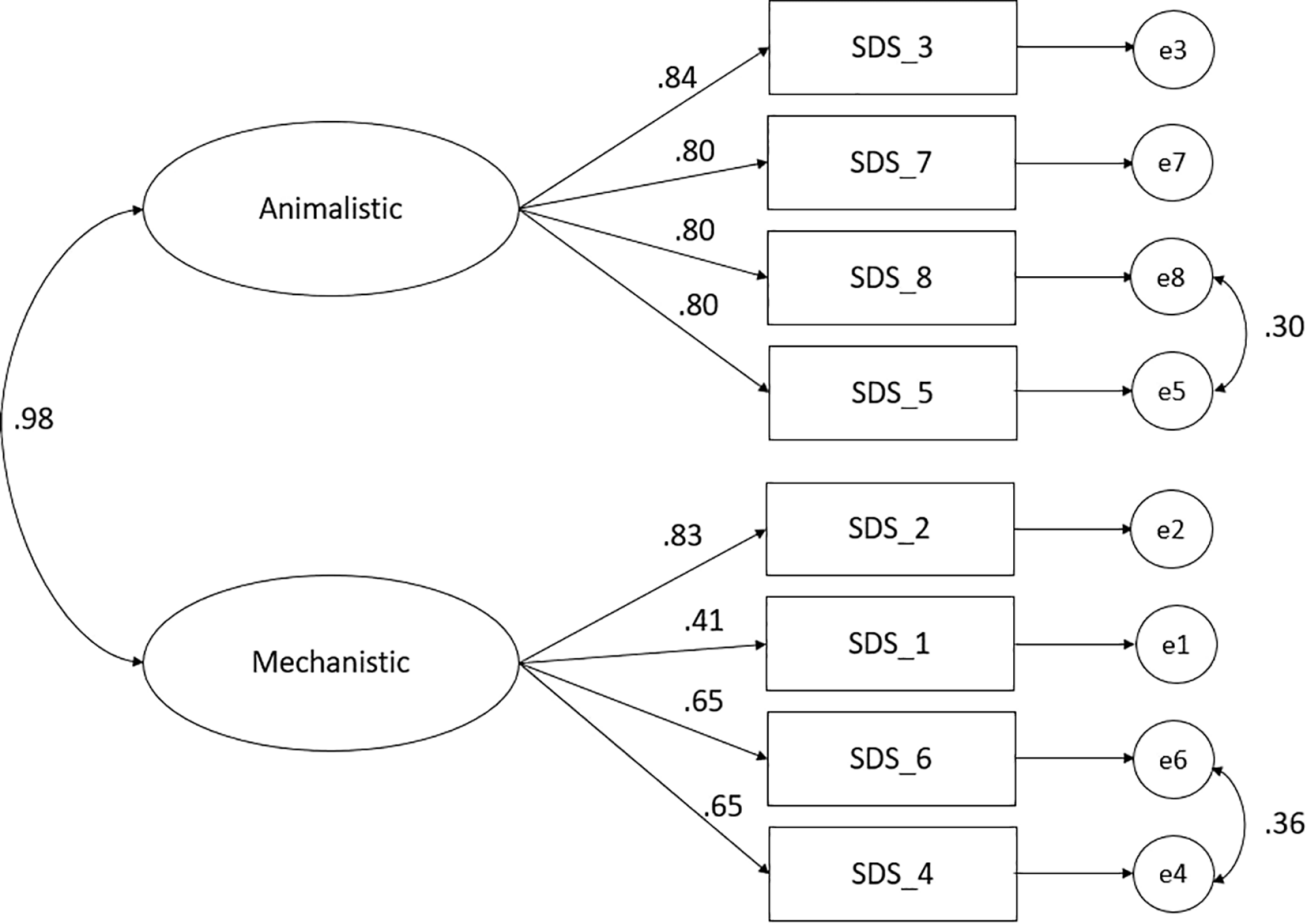
Optimized measurement model of the K-SDS. Straight arrows represent standardized regression weights, while curved arrows indicate correlations between the two sub-factors and between error terms.

In contrast, the correlated two-factor model, which aligned with the original SDS structure, provided the best fit among the initially tested models. The two-factor solution demonstrated an acceptable fit (χ²[17] = 111.17, p <.001; CFI = .976; TLI = .960; RMSEA = .081; SRMR = .030). Two error covariances were added based on the modification indices and semantic similarity of item content: Items 4 (“I sometimes feel mechanical and cold, like a robot”) with 6 (“I sometimes consider myself as an automaton”) and Items 8 (“I am a monster”) with 5 (“I am less evolved than most other humans”). The final two-factor model demonstrated an acceptable fit with all items loaded significantly on their respective factors (see **Figure 1**). For both subscales, the composite reliability values exceeded.70, supporting convergent validity (see **Table 3**) (38).

**Table 3:** Confirmatory factor analysis.

Factor/Item	*b*	*β*	S.E	C.R	CR
Animalistic Self-Dehumanization					**.89**
SDS-3	1.000	.841	–	–	
SDS-7	.887	.801	.033	27.209***	
SDS-8	.689	.801	.026	26.970***	
SDS-5	.818	.802	.030	27.042***	
Mechanistic Self-Dehumanization					**.74**
SDS-2	.942	.833	.047	20.082***	
SDS-1	.676	.406	.062	10.817***	
SDS-6	.944	.655	.045	20.824***	
SDS-4	1.000	.652	–	–	

*B*, unstandardized factor loading; *β*, standardized factor loading; S.E., standard error; C.R., critical ratio; CR, composite reliability; **p <.001.

The bold values represent the composite reliability (CR) coefficients for each latent factor.

The symbol *** indicates statistical significance at p < .001.

To further examine the dimensionality of the K-SDS and evaluate the appropriateness of reporting a total score, a bifactor model was additionally tested. The bifactor model demonstrated superior fit relative to the correlated two-factor model (χ²[12] = 64.33, p <.001; CFI = .987; TLI = .969; RMSEA = .072; SRMR = .021; AIC = 18223.22), whereas the correlated two-factor model yielded higher AIC values and lower incremental fit indices (AIC = 18400.39). These findings indicate the presence of a strong general self-dehumanization factor underlying the items while also supporting the distinctiveness of the animal self-dehumanization and mechanic self-dehumanization sub-dimensions.

### 3.3 Measurement invariance across sex

Measurement invariance was assessed across sex and educational level using a sequential procedure (configural, metric, scalar, and residual invariance). For both groups, all levels showed acceptable fit indices and changes within the recommended cut-offs (ΔCFI ≤.01; ΔRMSEA ≤.015), indicating full measurement invariance (see **Table 4**).

**Table 4:** Measurement invariance across sex.

Measurement invariance	*χ²*	*df*	TLI	CFI	RMSEA	SRMR	*δχ²*	*P*	ΔCFI
Configural invariance	203.41	38	.936	.957	.072	.054	–	–	–
Metric invariance	219.94	44	.941	.954	.069	.061	16.53	.011	.004
Scalar invariance	238.72	52	.947	.951	.065	.061	18.78	.016	.005
Residual invariance	268.76	61	.950	.946	.064	.065	30.05	.000	.008

Df, degrees of freedom; CFI, Comparative Fit Index; TLI, Tucker Lewis Index; RMSEA, Root Mean Square Error of Approximation; SRMR, Standardized Root Mean Square Residual.

### 3.4 Latent mean analysis of the K-SDS

Latent mean analysis was conducted by fixing the mean of the male group at zero and estimating the latent mean values for the female group. The results indicated that the mean difference between men and women on the mechanistic self-dehumanization factor was -0.17 (p = .032), corresponding to a minimal effect size. In contrast, women scored significantly lower on the animalistic self-dehumanization factor than men, with a mean difference of -0.25 (p <.001), reflecting a small effect.

Additionally, group differences were examined according to educational level (lower, middle, and higher education). Measurement invariance across educational levels was supported at all levels. Latent mean comparisons indicated that participants with higher education reported lower levels of both mechanistic and animalistic self-dehumanization than the lower and middle groups (see **Table 5**).

**Table 5:** Latent means of the K-SDS across education level groups.

Sub-factors	Lower education	Middle education	Higher education	Lower & middle *(d)*	Lower & higher *(d)*	Middle & higher *(d)*
Animalistic Self-Dehumanization	.050	0.000	-.394	.05	-.44	-39
Mechanistic Self-Dehumanization	-.036	0.000	-.218	-.04	-.18	-.22

The middle education group was set at zero. Negative values indicate lower latent mean values relative to the reference group. Cohen’s *d* values approximate standardized effect sizes, with .20, small, .50, medium; and .80, large.

### 3.5 Correlations between the K-SDS and other measures

The K-SDS total score was positively correlated with depression, suicidal ideation and behavior, thwarted belongingness, perceived burdensomeness, and self-hatred. Both the animalistic and mechanistic subscales showed significant positive correlations with these measures. However, the K-SDS was not correlated with fearlessness about death, supporting discriminant validity (see **Table 6**).

**Table 6:** Correlations between the KSDS and other measures (*N* = 844).

Measure	SDS_Total	SDS_A	SDS_M
PHQ-9	.49**	.48**	.48**
DSI-SS	.33**	.35**	.32**
INQ-10 TB	.48**	.49**	.47**
INQ-10 PB	.56**	.58**	.54**
FSCRS – Hated Self	.58**	.59**	.55**
ACSS-FAD	-.01	.00	-.02

SDS_A, Animalistic self-dehumanization; SDS_M, Mechanistic self-dehumanization; PHQ-9, Patient Health Questionnaire-9; DSI-SS, Depressive Symptom Inventory – Suicidality Subscale; INQ-10 TB, Interpersonal Needs Questionnaire – Thwarted Belongingness; INQ-10 PB, Interpersonal Needs Questionnaire – Perceived Burdensomeness; FSCRS-HS, Forms of Self-Criticism and Reassurance Scale – Hated Self; ACSS-FAD, Acquired Capability for Suicide Scale – Fearlessness About Death; *P* <.01.

The symbol ** indicates statistical significance at p < .01 (2-tailed).

### 3.6 Regression into independent variables

We performed multiple regression analyses to examine the associative power of the two K-SDS sub-factors, animalistic self-dehumanization and mechanistic self-dehumanization, on depression, suicidal ideation, self-hatred, thwarted belongingness, and perceived burdensomeness (see **Table 7**). Together, these two variables explained 24.8%, 12.6%, 56.2%, 25.4%, and 34.5% of the variance in depression, suicidal ideation, self-hatred, thwarted belongingness, and perceived burdensomeness, respectively.

**Table 7:** Multiple regression results for the K-SDS subscales predicting mental health outcomes (*N* = 844).

Outcome variable	Predictor	*B*	*SE*	*β*	*t*	*p*	*R²* (Adj. *R²*)
PHQ-9	SDS_A	0.307	0.065	.257	4.70	<.001	.248 (.246)
	SDS_M	0.311	0.065	.263	4.81	<.001	
DSI-SS	SDS_A	0.139	0.027	.299	5.07	<.001	.126 (.124)
	SDS_M	0.030	0.027	.065	1.11	.268	
FSCRS–HS	SDS_A	0.347	0.024	.606	14.52	<.001	.562 (.561)
	SDS_M	0.094	0.024	.165	3.95	<.001	
INQ-10 TB	SDS_A	0.498	0.048	.335	6.15	<.001	.254 (.252)
	SDS_M	0.276	0.080	.189	3.47	<.001	
INQ-10 PB	SDS_A	0.628	0.068	.417	9.40	<.001	.345 (.343)
	SDS_M	0.286	0.076	.192	3.76	<.001	

SDS_A, Animalistic self-dehumanization; SDS_M, Mechanistic self-dehumanization; PHQ-9, Patient Health Questionnaire-9 (depression); DSI-SS, Depressive Symptom Inventory – Suicidality Subscale (suicidal ideation); FSCRS-HS, Forms of Self-Criticism and Reassurance Scale – Hated Self (self-hatred); INQ-10 TB, Interpersonal Needs Questionnaire – Thwarted Belongingness; INQ-10 PB, Interpersonal Needs Questionnaire – Perceived Burdensomeness; Reported values are unstandardized coefficients (*b*), standard errors (SE), standardized coefficients (β), *t*-values, *p*-values, and explained variance (*R²*, adjusted *R²*).

Upon separate examination, SDS-A was found to be significantly associated with all five outcomes: depression, suicidal ideation, self-hatred, thwarted belongingness, and perceived burdensomeness. The SDS-M was significantly associated with depression, self-hatred, thwarted belongingness, and perceived burdensomeness but did not significantly correlate with suicidal ideation.

## 4 Discussion

The present study evaluated the psychometric properties of the Korean version of the Self-Dehumanization Scale (K-SDS) using a large sample. Overall, the results revealed that the K-SDS had strong internal consistency, supported the original two-factor structure, and provided evidence supporting its convergent, discriminant, and cross-group validity.

Similar to the original scale (20), the two-factor model of the K-SDS with the mechanistic and animalistic self-dehumanization domains demonstrated the best fit. In the original development study (20), the two-factor model showed excellent fit (CFI = .979; TLI = .969; RMSEA = .054; SRMR = .039). The present findings were consistent with these results, as the Korean version also demonstrated strong incremental fit indices (CFI = .976; TLI = .960) and acceptable residual-based indices (RMSEA = .081; SRMR = .030), supporting the stability of the factorial structure across cultural contexts. In contrast, the one-factor and higher-order models did not reach the recommended thresholds and were therefore not retained. Nevertheless, similar to the original validation study, it is meaningful to report the total scores because both the original and current studies have verified the reliability of the full scale, and existing research has consistently utilized total scores. In response to the substantial correlation between the two sub-dimensions, an additional bifactor analysis was conducted. The results further supported this interpretation, indicating the presence of a strong general self-dehumanization factor underlying the items while retaining the conceptual distinction between the mechanistic and animalistic domains. Thus, the findings support the two-factor model as the most appropriate representation of self-dehumanization, while also allowing the determination of a total score. Furthermore, reliability analyses indicated that both the sub-factors and the total scale exhibited good internal consistency.

While optimizing the model fit, we allowed correlated error terms between Items 4 and 6 and between Items 8 and 9 based on both modification indices and conceptual overlap. Specifically, Items 4 (“I sometimes feel mechanical and cold, like a robot”) and 6 (“I sometimes consider myself as an automaton”) have highly similar phrasing that may artificially inflate shared residual variance, while Items 8 (“I am a monster”) and 5 (“I am less evolved than most other humans”) capture extreme forms of animalistic dehumanization. These correlations were judged to reflect item content overlap rather than substantial deviations from the theoretical structure. The final model achieved a satisfactory fit after their inclusion. Nevertheless, correlated residuals may also reflect shared wording or potential redundancy between items and should therefore be interpreted with caution.

Although Item 1 (“I view myself as a number”) had the weakest factor loading, it remained statistically significant and conceptually aligned with the intended construct. Dehumanization is characterized by the dissolution of individuality, where people are perceived as indistinguishable and reduced to mere numbers (8, 39, 40). This aspect of mechanistic self-dehumanization would have been underrepresented if Item 1 had been excluded. Furthermore, self-dehumanization can stem from structural and individual factors, such as socioeconomic status (41) and numerical identification practices (42), both of which directly reflect the construct tapped by Item 1. Thus, despite its relatively weak loading, Item 1 provides a unique conceptual value and its retention helps represent the multifaceted experience of self-dehumanization in the K-SDS.

Additionally, the findings supported convergent and discriminant validity. The K-SDS total score and its sub-factors were positively associated with depression (PHQ-9), suicidal ideation (DSI-SS), thwarted belongingness, perceived burdensomeness (INQ-10), and self-hatred (FSCRS-HS). Notably, it was strongly correlated with self-hatred, underscoring the central role of internal self-criticism in self-dehumanization (43). In contrast, the correlations with the ACSS-FAD (fearlessness about death) were not significant, supporting discriminant validity. This finding is consistent with Joiner’s (18) interpersonal theory of suicide, which emphasizes that fearlessness about death is more closely related to the enactment of suicidal behavior than suicidal ideation. Therefore, these results suggest that self-dehumanization is closely associated with affective and interpersonal vulnerabilities rather than acquired capabilities.

Furthermore, latent mean analysis revealed significant group differences. Women reported significantly lower levels of animalistic self-dehumanization than men, although no sex differences were observed in mechanistic self-dehumanization. Furthermore, educational level played a significant role; individuals with higher educational levels reported lower overall self-dehumanization. This suggests that education may function as a protective factor against the internalization of dehumanization experiences.

Unlike the original study, which found mechanistic self-dehumanization to be a strong predictor of depression and suicidal ideation, the present study demonstrated that animalistic self-dehumanization was strongly associated with these outcomes. Cross-cultural considerations may provide further insight into these findings. Previous studies have emphasized that human uniqueness (HU)—attributes such as civility, morality, and refinement—play a central role in East Asian cultural contexts (1, 9, 44). In the present study, the strong links between animalistic self-dehumanization and depression/suicidal thoughts may reflect the high value placed on HU characteristics in Korean society. Therefore, perceiving oneself as lacking refinement or morality may be especially distressing in this cultural setting, thereby highlighting animalistic self-dehumanization as a significant predictor of psychological risk. However, these cultural interpretations should be considered theoretical rather than empirically tested explanations, as cultural variables were not directly assessed in the present study.

### 4.1 Limitations

Despite these strengths, this study had several limitations. First, the cross-sectional design restricted the ability to determine causal or temporal relationships between self-dehumanization and psychological difficulties. Longitudinal and experimental designs are necessary to determine causal relationships. Second, the exclusive use of self-report measures introduces potential biases, such as social desirability or self-perception distortions. Furthermore, because all data were collected via a single survey, common method variance cannot be ruled out and may have inflated associations between constructs. Future studies should employ multiple-method approaches to mitigate this concern. Additionally, as test–retest reliability was not assessed, the temporal stability of the K-SDS remains to be established. Third, the sample was limited to community-dwelling adults recruited online, which reduces the generalizability of the findings to clinical populations or adolescents. Fourth, although the results supported the two-factor structure, additional cross-cultural validation is necessary to confirm whether animalistic and mechanistic self-dehumanization function similarly across cultures. Finally, the interpretation of animalistic items requires caution, as animals may carry positive symbolic meanings in certain cultural contexts.

## 5 Conclusion

The Korean version of the Self-Dehumanization Scale demonstrated good psychometric properties, highlighting its reliability and validity for assessing self-dehumanization among Korean adults. Notably, the findings highlighted the role of animalistic self-dehumanization in depression and suicidal ideation, suggesting its salience in East Asian contexts that emphasize human uniqueness. Thus, the K-SDS is a valuable instrument for evaluating self-dehumanization and its implications for psychological well-being and suicide risk in both research and clinical settings.

## Data Availability

The raw data supporting the conclusions of this article will be made available by the authors, without undue reservation.
