# Supplementary material for: Validation of the Korean version of the Self-Dehumanization Scale
Source: Front Psychiatry. 2026 Mar 5;17:1754166. doi: 10.3389/fpsyt.2026.1754166 (PMC13000390; doi:10.3389/fpsyt.2026.1754166)
Supplement: Supplementary file 1 [file Table1.docx]

Supplementary Material

# Appendix 1. K-SDS Items

## o 아래 각 항목을 읽고, 해당 문장이 자신을 얼마나 잘 설명하는지 평가해 주세요

| 번호 | 문항 | 전혀 동의하지 않음 | 거의 동의하지 않음 | 약간 동의하지 않음 | 보통임 | 약간 동의함 | 거의 동의함 | 완전히 동의함 |
| --- | --- | --- | --- | --- | --- | --- | --- | --- |
| 1 | 나는 숫자로 인식되는 존재이다.(예: 학번) | ① | ② | ③ | ④ | ⑤ | ⑥ | ⑦ |
| 2 | 나는 나 자신을 무생물이라고 생각한다.(예: 살아숨쉬는 존재가 아닌 것 같다.) | ① | ② | ③ | ④ | ⑤ | ⑥ | ⑦ |
| 3 | 나는 내 자신을 사람보다 모자란 존재로 느껴서 사회적으로 고립되어 있다. | ① | ② | ③ | ④ | ⑤ | ⑥ | ⑦ |
| 4 | 나는 때때로 나에게서 로봇 같은 기계적이고 냉담한 느낌을 받는다. | ① | ② | ③ | ④ | ⑤ | ⑥ | ⑦ |
| 5 | 나는 대부분의 다른 사람들보다 덜 진화했다. | ① | ② | ③ | ④ | ⑤ | ⑥ | ⑦ |
| 6 | 나는 때때로 나 자신을 자동화된 기계라고 여긴다. | ① | ② | ③ | ④ | ⑤ | ⑥ | ⑦ |
| 7 | 나는 나의 인긴답지 못한 면 때문에 내 자신이 혐오스럽다. | ① | ② | ③ | ④ | ⑤ | ⑥ | ⑦ |
| 8 | 나는 괴물이다. | ① | ② | ③ | ④ | ⑤ | ⑥ | ⑦ |
